# The Preliminary Study of Effects of Tolfenamic Acid on Cell Proliferation, Cell Apoptosis, and Intracellular Collagen Deposition in Keloid Fibroblasts *In Vitro*

**DOI:** 10.1155/2014/736957

**Published:** 2014-09-22

**Authors:** Dan Yi, Ji Bihl, Mackenzie S. Newman, Yanfang Chen, Richard Simman

**Affiliations:** ^1^Department of Pharmacology and Toxicology, Boonshoft School of Medicine, Wright State University, 3640 Colonel Glenn Hwy, Dayton, OH 45435, USA; ^2^Department of Plastic and Reconstructive Surgery, Boonshoft School of Medicine, Wright State University, 3640 Colonel Glenn Hwy, Dayton, OH 45435, USA

## Abstract

Keloid scarring is a fibroproliferative disorder due to the accumulation of collagen type I. Tolfenamic acid (TA), a nonsteroidal anti-inflammatory drug, has been found to potentially affect the synthesis of collagen in rats. In this preliminary study, we aimed to test the effects of TA on cell proliferation, cell apoptosis, and the deposition of intracellular collagen in keloid fibroblasts. Normal fibroblasts (NFs) and keloid fibroblasts (KFs) were obtained from human dermis tissue. Within the dose range 10^−3^–10^−6^ M and exposure times 24 h, 48 h, and 72 h, we found that 0.55 × 10^−3^ M TA at 48 h exposure exhibited significantly decreased cell proliferation in both NFs and KFs. Under these experimental conditions, we demonstrated that (1) TA treatment induced a remarkable apoptotic rate in KFs compared to NFs; (2) TA treatment reduced collagen production in KFs versus NFs; (3) TA treatment decreased collagen type I expression in KFs comparing to that of NFs. In summary, our data suggest that TA decreases cell proliferation, induces cell apoptosis, and inhibits collagen accumulation in KFs.

## 1. Introduction

Keloid scarring, is a raised scar which forms by expanding beyond the boundaries of the original lesion [1]. The main histological manifestation of a keloid scar is the overgrowth of atypical fibroblasts with excessive accumulation of extracellular matrix components, especially collagen, fibronectin, elastin, and proteoglycans [1–4]. The causes of this type of scar are still unknown, but it has been pointed out that keloid scars can develop after any dermal abrasion including burns, piercing, or surgery [1–6]. Differing from normal wound healing, keloid scar formation begins with abnormal tissue growth in the dermal lesion extending beyond the borders of the original wound [7–11]. The central pathological wound healing response of keloid scarring is composed of a high density of mesenchymal cells called keloid fibroblasts (KFs) [9, 10]. Consequently, the over growth of KFs results in overabundance of extracellular and intracellular matrix stroma, which is classified by irregularly directed and thick hyalinized spiral bundles described as keloidal collagen [5, 12]. During the formation of keloid scars, the type of collagen initially secreted by fibroblasts is granular collagen type III. Throughout the maturation of the process, collagen type I gradually replaces collagen type III and eventually comprises extracellular matrix in 99% of the wound bed [1, 5, 9–12]. Current, common treatments for keloids are often a combination of excision followed by a reconstructive surgical procedure. Glucocorticoids or 5-fluorouracil injections followed by compression therapy such as silicone sheets are frequently employed [1, 2]. Nonetheless, recurrence remains between 45% and 100% [3, 4]. Therefore, treatment of keloids continues to be a great challenge for the reconstructive surgeon.

**Figure 1:**
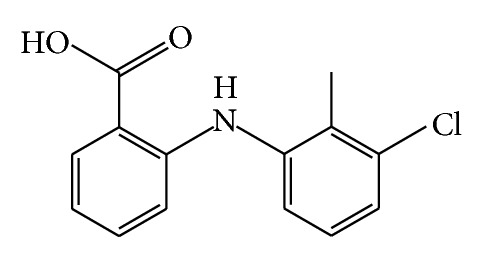
Structure of tolfenamic acid.

Tolfenamic acid (TA) is a fenamic acid derivative belonging to the nonsteroidal anti-inflammatory drug (NSAID) class that is traditionally used for rheumatic diseases [13, 14]. The predominant medical uses of this group of drugs include rheumatoid arthritis, osteoarthritis, and inflammatory arthropathies [15–18]. TA, also written as 2-([3-chloro-2-methylphenyl]-amino)-benzoic acid in IUPAC terms (Figure 1), has a low solubility in water and molecular weight of 261.7 g/mol. The exact medical applications, adverse effects, and mechanism of TA are not clear. However, previous studies have described highly specific applications of TA. Studies illustrate that TA is associated with inhibiting collagen metabolism in connective tissue in rats and has the capacity to induce cancer cell apoptosis [13, 14, 19–25]. There is an inhibition of sodium tolfenamate on the metabolism of collagen with 0.15 mol/L NaCl in rats [13]. TA reduces cell survival, growth, and angiogenesis in tumor and cancer cells, including human xenograft tumor, human pancreatic cancer, human neuroblastoma, and mouse prostate cancer by regulating the activity of transcription factor Sp1; human head and neck cancer by regulating NADAG-1; human colorectal cancer via ESE-1/EGR-1; and human oral cancer by affecting the p38 mitogen-activated protein kinase signaling pathway [14, 19–23].

## 2. Materials and Methods

### 2.1. Cell Culture and Chemicals

All skin samples were obtained under Wright State University IRB number SC4833. A sample of scar tissue (KF1) was taken from a 24-year-old African-American male with clinical and pathologic evidence of keloid scarring confirmed as previously described [10, 26]. A second keloid fibroblast cell line (KF2) was obtained from a 35-year-old African-American female was purchased from ATCC (Passage 11, ATCC, USA). One normal adult skin sample (NF) was obtained from a 29-year-old African-American female during plastic surgery [7, 8]. Skin specimens were incubated with 2 mL digestion medium containing high glucose Dulbecco's Modified Eagle's Medium (DMEM) (Gibco, Life Technologies, USA), 5 mg/mL collagenase/dispase II (RocheDiagnostics, USA), and 0.25% trypsin (Invitrogen, Life Technologies, USA) for 8 hours under 5% CO_2_, at 37°C [7, 9, 27]. Isolated fibroblasts passages (P) 0 from keloid scar tissue and normal dermis tissue were cultured in total medium comprising of high glucose DMEM, 10% fetal bovine serum (Gibco, Life Technologies, USA), 1% pen/strep/glutamine (Invitrogen, Life Technologies, USA) in the condition of 5% CO_2_, at 37°C. Each cell line was cultured separately. KF1 P13 to P15, KF2 P 3 to 5, and NF P3–P8 were tested in all assays. As both KF1 and KF2 were conducted in each assay, generally it will refer to KF in the following. TA was purchased from Cayman Chemical Company, USA.

### 2.2. MTT

NFs and KFs were divided into different groups for pretreatment with TA (10^−3 ^M, 10^−4 ^M, 10^−5 ^M, or 10^−6 ^M) for different periods (24 h, 48 h, or 72 h), respectively. An MTT kit (Invitrogen, Life Technologies, USA) was used for the proliferation assay. Absorbance was detected at 535 nm in a Packard Fusion spectrophotometer [27]. Relatives of Cell proliferation rate (%) were determined with cell cultures and analyzed according to the following equation:

(1) Fibroblast  proliferation  rate  (%) =OD535  (TA  treatment  group)OD535  (Non-TA  treatment  group).

All experiments were performed independently. Six times with keloid fibroblast cultures culture as well as normal skin dermal culture.

### 2.3. Apoptosis

NFs and KFs were divided into three groups Control (“C”, only treated with medium), Vehicle (“V”, medium 0.55% DMSO), and TA (550 *μ*M TA dissolved in 0.55% DMSO with medium) for 48 h exposure separately. Annexin-binding buffer (Invitrogen, Life Technologies, USA) and propidium iodide (Invitrogen, Life Technologies, USA) working solutions were prepared at 1X. Cell apoptosis were detected by a fluorescence-activated cell sorting (FACS) Calibur Flow Cytometer (Accuri C6, Inc., USA) [20, 21]. All experiments were performed independently. Four times with keloid fibroblast cultures culture as well as normal skin dermal culture.

### 2.4. Collagen Staining

NFs and KFs were divided into three groups Control (“C”, only treated with medium), Vehicle (“V”, medium 0.55% DMSO), and TA (550 *μ*M TA dissolved in 0.55% DMSO with medium) for 48 h exposure separately. Sirius red/Fast green staining kit (Chondrex, USA) was used to analyze collagen production. Absorbances at 535 nm and 600 nm were detected by a Packard Fusion spectrophotometer and analyzed by the following equation:

(2) $$\begin{array}{c} \text{Collagen}\;\left(\mu \text{g}/\text{well}\right)=\frac{\text{OD}535-0.291*\text{OD}600}{0.037}. \end{array}$$

All experiments were performed independently. Four times with keloid fibroblast cultures culture as well as normal skin dermal culture.

### 2.5. Western Blot

NFs and KFs were divided into three groups Control (“C”, only treated with medium), Vehicle (“V”, medium 0.55% DMSO), and TA (550 *μ*M TA dissolved in 0.55% DMSO with medium) for 48 h exposure separately. Proteins were extracted from each cell line and the concentrations were measured by BCA assay. Sixty micrograms of each sample were mixed with 10 *μ*L of 5X protein sample loading buffer. The trans-blotted PVDF membrane was then blocked with blocking buffer (3% BSA; 1X TBS; and 0.05% Tween-20) for 1 h. Afterward, It was incubated with rabbit monoclonal anti-human collagen type I (dilution 1 : 2000, Thermo Fisher Scientific, USA), over two nights at 4°C. Anti-rabbit HRP-conjugated secondary antibody (dilution at 1 : 20000, Sigma, USA) was then added to the membrane on a shaker for an hour at room temperature. After rinsing with TBST, the membrane was activated with chemiluminescent HRP substrate (Cell Signaling Technology, USA) for 4 min in the dark at room temperature. It was visualized and quantified using a chemiluminescent detection system (Bio-Rad, USA). Protein band intensity in each lane was scored by volume intensity and was normalized to beta-actin (dilution at 1 : 4000, Sigma, USA) [27]. All experiments were performed independently. Four times with keloid fibroblast cultures culture as well as normal skin dermal culture.

### 2.6. Statistics

Statistical analysis was performed with STATISTICA version 6.0 (StatSoft, Inc. USA). Data are displayed as mean ± SEM which was evaluated by one-way ANOVA between two groups in this study. *P* < 0.05 was considered statistically significant for all tests.

## 3. Results

### 3.1. Dose- and Time-Response of TA on NFs and KFs Cell Proliferation

**Figure 2:**
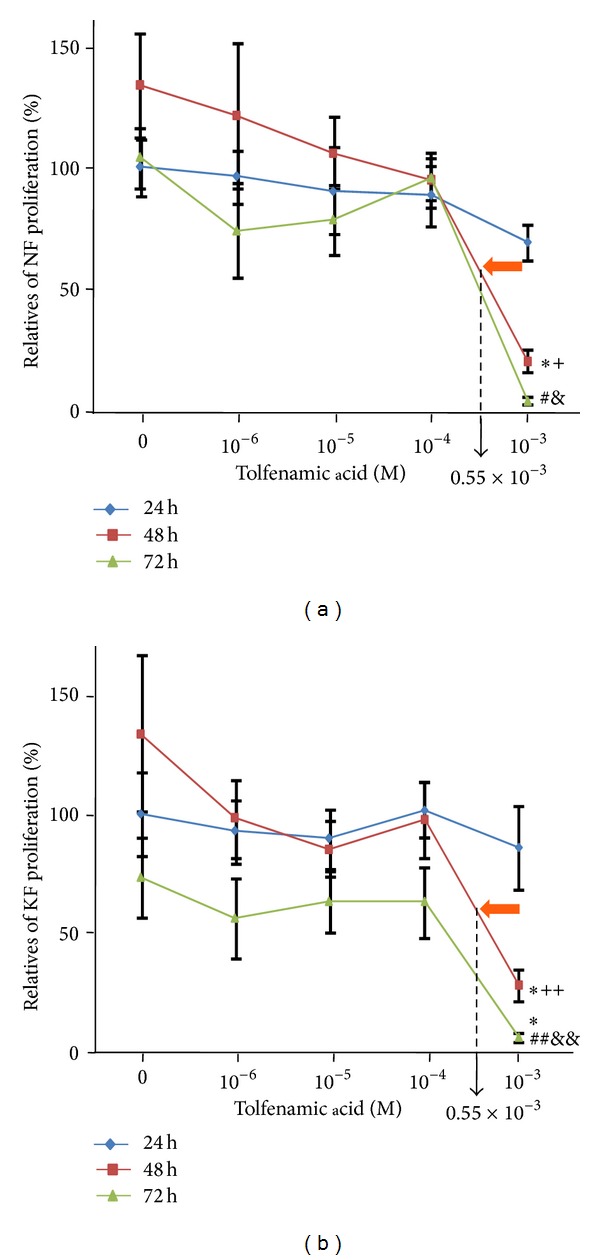
The dose- and time-responses of TA on cell proliferation in NFs and KFs. In 48 h exposure time group and 72 h exposure time group, 10^−3 ^M TA significantly decreased cell proliferation in NFs. 0, 10^−6^, 10^−5^, 10^−4^, 10^−3^, 0.55 × 10^−3^: M TA was dissolved in 1% DMSO. *P < 0.01, 10^−3 ^M versus 0 M; ^+^ *P* < 0.01, 48 h versus 24 h; ^#^ *P* < 0.01, 10^−3 ^M versus 0 M or 10^−4 ^M; ^&^ *P* < 0.01, 72 h versus 24 h or 48 h; **P < 0.01, 10^−3 ^M versus 0 M; ^++^ *P* < 0.05, 48 h versus 24 h; ^##^ *P* < 0.05, 10^−3 ^M versus 0 M; ^&&^ *P* < 0.01, 72 h versus 24 h or 48 h; *n* = 6.

The effect of TA on NFs and KFs proliferation was evaluated by MTT analysis and this is displayed in Figure 2. At the highest concentration of TA, cell proliferation rates appeared to decrease. As the exposure time increased, NF and KF proliferation sharply decreased at 10^−3 ^M TA. Cell proliferation also decreased at a greater rate when TA was applied over 48 or 72 hours. Based on the results of this assay, 0.55 × 10^−3 ^M TA at 48 h exposure was determined to be the ideal condition for future experimentation.

### 3.2. The Effect Dose and Time of TA on Inducing Cell Apoptosis in KFs

**Figure 3:**
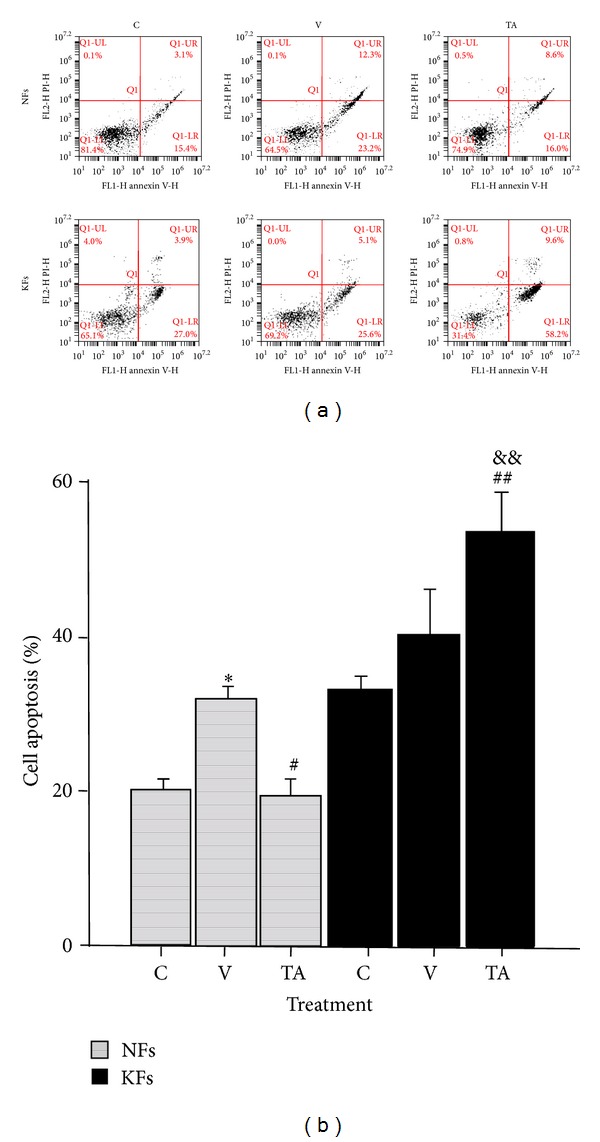
The effect of TA on cell apoptosis in NFs and KFs. DMSO induced early and late apoptosis in NFs and slightly induced early apoptosis in KFs. TA strikingly induced late apoptosis in KFs. C: control, total medium; V: vehicle, 0.55% DMSO; TA, 0.55 × 10^−3^ M of TA was dissolved in 0.55% DMSO. *P < 0.01, V versus C or TA; ^#^ *P* < 0.05, TA versus V; ^##^ *P* < 0.05, TA versus C; ^&&^ *P* < 0.01, TA versus TA; *n* = 6.

To determine whether TA induced cell apoptosis on NFs and KFs, Annexin-V/PI labeling was administered after applying 0.55 × 10^−3 ^M TA dissolved in 0.55% DMSO for 48 h. Although the vehicle alone exhibited an effect on NFs, selectively significant apoptosis in TA-treated KFs compared with non-TA treatment groups of KFs and in NFs (Figure 3).

### 3.3. The Effect Dose and Time of TA on Inhibiting Collagen Accumulation in KFs

**Figure 4:**
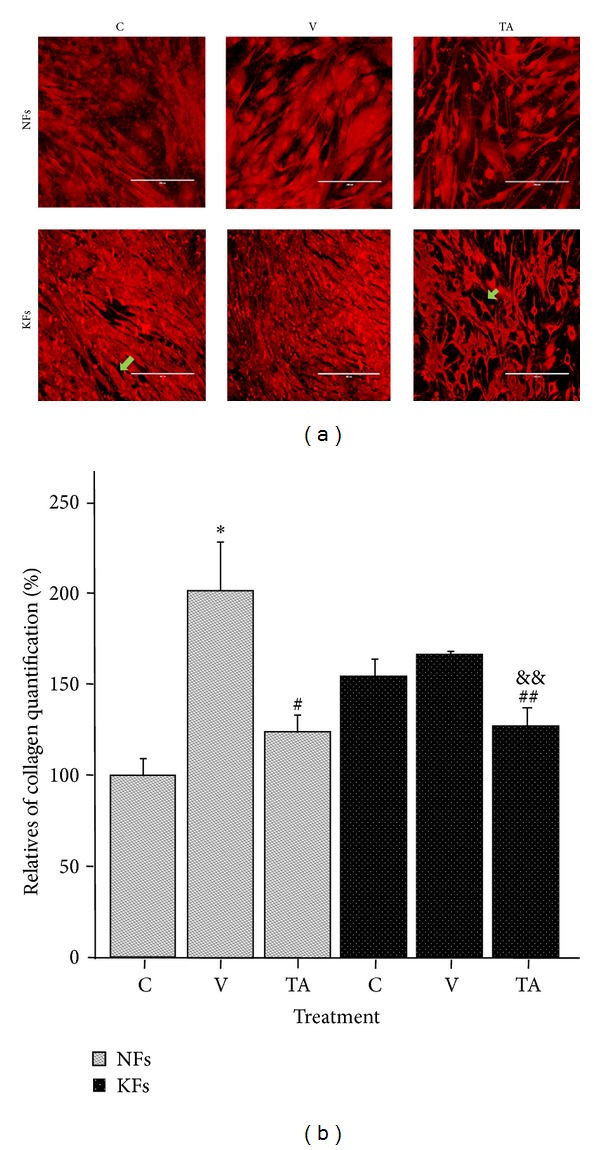
The effect of TA on collagen production in NF and KF after Sirius red staining. DMSO significantly increased collagen production in NFs. TA strikingly reduced collagen production in KFs compared with NFs. C: control, total medium; V: vehicle, 0.55% DMSO; TA, 0.55 × 10^−3 ^M of TA was dissolved in 0.55% DMSO. *P < 0.01, V versus C; ^#^ *P* < 0.05, TA versus V; ^##^ *P* < 0.05, TA versus C; ^&&^ *P* < 0.01, TA versus V; *n* = 4.

Quantitative analysis of intracellular and extracellular Sirius red fluorescent staining reflects the degree of all types of collagen produced by fibroblasts. Results indicated that collagen production was significantly reduced in KFs at the previously determined dose and time (Figure 4). Additionally, there was a considerable morphological change in KFs. Stained intensity decreased in all samples. Secreted, stained proteins and cell density were both reduced, especially in KF cultures.

### 3.4. The Effect Dose and Time of TA on Reducing the Expression of Collagen Type I in KFs

**Figure 5:**
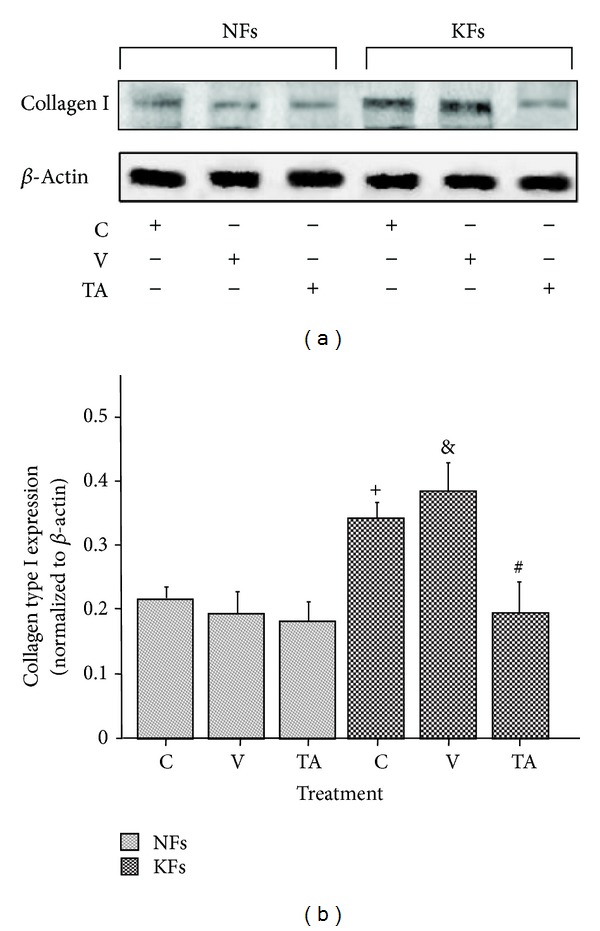
The effect of TA on the expression of collagen type I in NFs and KFs. DMSO slightly increased the expression of collagen type I in KFs. TA significantly inhibited the expression of collagen type I in KFs. C: control, total medium; V: vehicle, 0.55% DMSO; TA, 0.55 × 10^−3 ^M of TA was dissolved in 0.55% DMSO. ^+^ *P* < 0.05, C versus C; ^&^ *P* < 0.05, V versus NFs C; ^#^ *P* < 0.05, TA versus V; *n* = 4.

The effects of TA on collagen type I (Collagen I) protein expression were evaluated by Western blot in both cell types. Protein quantity was measured 48 hours after each treatment.  Figure 5 shows a strong increased expression in KFs compared to NFs. TA significantly suppressed the expression of collagen type I in KFs compared with the other groups, while the inhibition of collagen type I expression in NFs after TA treatment was not significant. This result demonstrated that 0.55 × 10^−3 ^M TA for 48 h inhibits collagen I expression significantly in KFs but not, effectively in NFs.

## 4. Discussion

Keloid scaring, a type of scars, is a fibroproliferation disease with the accumulation of collagen deposition caused by the upregulation of autocrine TGF-*β* signaling during the wound healing process [5, 13, 27]. The role of TA, a fenamate and NASID, has been used recently for cancer treatments [13, 14, 19–25]. These experiments were designed to determine if the administration of TA is able to alter the proliferatory rates of keloid fibroblasts and decrease collagen production in KFs. Our preliminary study demonstrates that TA has the potential to normalize some of the characteristic features of KFs such as cell apoptosis and collagen production.

Initially, we tested the effects of TA on NF and KF proliferation by applying five concentrations of TA (10^−3 ^M, 10^−4 ^M, 10^−5 ^M, and 10^−6 ^M) for 3 different exposure times (24 h, 48 h, and 72 h) separately in order to determine the conditions at which minimal drug concentration had the greatest proportionate effect on disease fibroblasts. We found that NF and KF proliferation rates were decreased with the 10^−3 ^M TA after 48 h exposure. This indicated that the diversity of NF and KF proliferation were significantly reduced by certain higher amount of TA. Linear extrapolation of cell proliferation assay, data at 48 h revealed that the effective concentration of TA which can result in half reduction of NF proliferation was 550 *μ*M. This concentration of TA at 48 h exposure did not show a significant reduction in KFs proliferation, but other effects were still expected to be present. At 72 hours, there was a large reduction in proliferation of KF cultures versus NFs. This may be due to a long-phase response to TA toxicity in KF or a side effect of changing culture media every three days. To further qualify the effects of this TA does and time exposure treatment, we conducted cell apoptosis assays with 0.55 × 10^−3^ M TA and with 48 h time exposure. A group exposed to 0.55% DMSO in medium was added to control for vehicle effects. Though 0.55% DMSO induced a proportional apoptotic rate in NFs and KFs, cell apoptosis in KFs after TA treatment induced a selectively significant apoptotic rate compared to NFs. This indicated that cell apoptosis was significantly influenced by TA. According to the typical histological changes in KFs [1–4], reducing collagen production is a primary pharmacological target to treat keloid scarring. In our study, we tested the effects of TA on decreasing the accumulation of collagen and furthermore tried to verify a relationship between cell apoptosis and collagen deposition. Based on our data, a high inhibition rate of collagen production was detected in KFs after 0.55 × 10^−3 ^M TA with 48 h exposure time compared with NFs. Although the effect of 0.55% DMSO increased collagen production in NFs, the production caused by 0.55% DMSO did not show specific significance in KFs. Concurrently, after TA treatment, cell number also decreased. Therefore, TA reduced collagen production in KFs with high efficiency. The Sirius red collagen quantification assay targets the helical collagen repeat bundles and is therefore nonspecific for specific types of collagens. The majority of collagen produced by KFs is collagen type I, and our results were specific for collagen type I after TA treatment [1, 2, 12, 26–28]. Our study confirms that collagen I is overexpressed in KFs versus NFs. Treatment with TA* in vitro* significantly decreased collagen expression in KFs over NFs.

In the cell apoptosis and collagen expression experiments, DMSO vehicle alone did have some effects compared to control group. The underlying mechanism for this is not apparent from our study. In cell apoptosis assays, compared with the treatment group, the vehicle group had more significant effect in NFs while the effect in KFs is not significant. This may be further considered for clinical studies. In collagen expression assays, DMSO did not significantly induce collagen expression in NFs. In KFs, it significantly increased the expression of collagen but this effect can be reversed by TA. In summary, DMSO did not influence the performance of TA on cell apoptosis and collagen expression in KFs. The present study is limited by the number of samples. Nonetheless, statistically significant effects were seen from TA treatment in KF cells. A potential mechanism for the activity of TA in keloid is its capacity to induce degradation of the Sp2 transcription factor. Sp1 has a role in the canonical TGF-*β* transduction pathway. Because TGF-*β* is widely regarded as the central progenitor of fibrotic scars and has a large capacity for autocrine signaling, the degradation of Sp1 may serve as a putative pharmacological target in keloids. Future studies may include the use of TGF-*β* receptor blockers and measuring intracellular phosphorylated SMAD family members. Other studies may include work to refine the dosage with respect to solubility without a vehicle. Although a genetic mouse model does not yet exist for keloids, there are still valid models of keloid and hypertrophic scarring that may function to validate the mechanism and activity of TA in this disease. Despite the shortcomings of this preliminary study, TA has clear potential to selectively treat keloid fibroblasts over normal dermal fibroblasts. Numerous other small-molecule treatments have been tested in keloids, but TA and fenamates on the whole, have not been explored.

Generally speaking, our current novel data demonstrated that tolfenamic acid induced cell apoptosis and inhibited collagen production in keloid fibroblasts. TA could be the new therapeutical application for treating keloid scars. With the development of the advanced technologies, hundreds and thousands of treatments on keloid scar have been reported. However, this is the first time where TA was successfully used* in vitro* to induce cell apoptosis and reduce collagen accumulation in KFs. Furthermore, TA is an available commercial formulation chemical. Thereby, TA is recommended for clinical trials to confirm our findings.
